# Microvillus in LBW Meishan Piglets Preserved Microvillus Integrity Alongside Impaired Intestinal Barrier Function in Low-Birth-Weight Meishan Neonatal Piglets

**DOI:** 10.3390/ani15213085

**Published:** 2025-10-24

**Authors:** Li Dong, You Wu, Zhixuan Sun, Hongrong Wang, Lihuai Yu

**Affiliations:** 1 Department of Animal Science and Technology, Yangzhou University, No.48 of East Wenhui Road, Yangzhou 225009, China; donglijiayou@126.com (L.D.); er120934@163.com (Y.W.); MZ120241634@stu.yzu.edu.cn (Z.S.); hrwang@yzu.edu.cn (H.W.); 2 Institute of Animal Nutrition, Sichuan Agricultural University, Chengdu 611130, China

**Keywords:** antioxidant, barrier function, intestine, low birth weight, Meishan piglets

## Abstract

**Simple Summary:**

The aim of this study was to evaluate the impact of low birth weight on intestinal barrier function in Meishan neonatal piglets and to compare structural and functional parameters between low-birth-weight (LBW) and normal-birth-weight (NBW) individuals. These parameters were classified into categories reflecting intestinal health status based on expert knowledge. Most structural and functional indicators were impaired in LBW piglets, accounting for significant alterations in barrier integrity, though microvillus structure remained notably intact. Negatively affected aspects included villus morphology, antioxidant capacity, and tight junction protein expression. A subsequent analytical approach using morphological, molecular, and biochemical assays was performed to objectively quantify these differences. Establishing an expert-based assessment of intestinal development is important for advancing strategies aimed at improving neonatal piglet survival. It forms the basis for detecting critical physiological deficits through biomarker analysis and supporting targeted interventions via precision husbandry.

**Abstract:**

Despite lower birth weight, Meishan piglets exhibit a notably higher pre-weaning survival rate compared to Western commercial breeds. This study aimed to evaluate the effect of low birth weight (LBW) on intestinal barrier function in Meishan neonates. Six pairs of neonatal piglets (one normal birth weight, NBW: 0.85 ± 0.06 kg; one LBW: 0.65 ± 0.02 kg) from the same sow were euthanized at birth prior to suckling. Morphological parameters, goblet cell density, antioxidant enzyme activities, cytokine gene expression, and tight junction protein levels in the small intestine (SI) were assessed. Results showed that LBW piglets had a significantly higher SI length-to-body weight ratio (*p* < 0.05), along with reduced villus height, villus height-to-crypt depth ratio, and villus surface area in the jejunum and ileum (*p* < 0.01). Notably, microvillus structure remained intact despite the presence of mitochondrial swelling. LBW piglets also exhibited decreased goblet cell numbers, lower antioxidant capacity, dysregulated expression of cytokines (CD8, IFNγ, IL4, IL2), and reduced levels of mucin 2, ZO-1, and occludin (*p* < 0.05). In conclusion, although LBW Meishan piglets showed impairments in multiple aspects of intestinal barrier function, the structural integrity of the microvillus was preserved, which may contribute to their higher survival rate and represents a key adaptive advantage over commercial pig breeds.

## 1. Introduction

Pre-weaning mortality remains a significant challenge in pig production, particularly in Western breeds, where low birth weight (LBW) is a major contributing factor [1,2]. Meishan piglets have a pre-weaning mortality rate of ~10–15%, significantly lower than the 20–30% typically observed in Western commercial breeds like Duroc × (Landrace × Yorkshire) [3]. Interestingly, Meishan piglets exhibit markedly lower pre-weaning mortality despite their reduced birth weight, suggesting unique physiological or genetic adaptations [3,4]. Our previous work demonstrated that Meishan neonates possess superior intestinal barrier function compared to Duroc × (Landrace × Yorkshire) crossbred piglets, which may partly explain their enhanced survival [5].

The intestinal barrier plays a vital role in nutrient absorption, immune defense, and protection against pathogens. Its key structural elements include villi, microvilli, goblet cells, tight junction proteins, and antioxidant systems. In commercial pig breeds, these elements can be compromised by LBW or intrauterine growth restriction (IUGR) [6,7]. However, whether LBW imposes similar detrimental effects on intestinal barrier function in Meishan piglets remains unclear.

Notably, the microvillus layer, which greatly amplifies the intestinal absorptive surface, is often disrupted in LBW neonates of conventional breeds, leading to impaired nutrient digestion and uptake [8]. The preservation of microvillus layer could be a critical factor mitigating the effects of LBW. This study is the first to comprehensively investigate the influence of LBW on the intestinal barrier in Meishan piglets, with particular emphasis on microvillus integrity, antioxidant capacity, mucosal immunity, and junctional complex proteins. We hypothesize that the structural and functional preservation of the microvillus contributes significantly to the resilience of Meishan piglets despite lower birth weight.

## 2. Materials and Methods

### 2.1. Animals and Experimental Design

Six pairs of newborn Meishan piglets were obtained from three sows at the Kunshan Meishan Pig Breeding Company (Kunshan, Jiangsu, China). Each pair consisted of one normal-birth-weight (NBW) and one low-birth-weight (LBW) piglet from the same sow. Healthy sows (parity 3–4) were managed and vaccinated under standard farm protocols. The sows were fed the same diet formulated according to the company’s standards (Table 1). Piglets with birth weights of 0.85 ± 0.06 kg and 0.65 ± 0.02 kg were classified into NBW and LBW group (3 male and 3 female in each group), respectively. A total of 12 neonatal piglets (6 NBW and 6 LBW) were euthanized via intramuscular injection of sodium pentobarbital (50 mg/kg body weight) 2–4 h after birth, prior to suckling.

### 2.2. Sample Collections

The small intestine (SI) was excised and segmented into the duodenum, jejunum, and ileum. After removing intestinal contents and mesenteric attachments, the length and weight of each segment were recorded. Approximately 1 cm segments from each region were collected and fixed in 4% buffered formaldehyde. Additionally, two samples from the midpoint of the SI were fixed in 2.5% buffered glutaraldehyde. After being rinsed with saline, the intestinal mucosa was scraped using a sterile glass slide. Mucosal tissues from the jejunum and ileum were then snap-frozen in liquid nitrogen and stored in a −80 °C freezer for further analysis.

### 2.3. Histological Analysis

Samples fixed in glutaraldehyde were processed for scanning and transmission electron microscopy using standard protocols [9]. Tissue samples were fixed, washed with 0.1 M PBS, and post-fixed with 1% osmium tetroxide. Subsequently, they were dehydrated through a graded ethanol series (30–100%), critical-point dried, and sputter-coated with a gold layer prior to imaging using a ZEISS GeminiSEM 300 microscope (Carl Zeiss AG, Jena, Germany) [10]. Samples were post-fixed with 1% osmium tetroxide for 2 h in the dark, dehydrated in an ethanol/acetone series, and embedded in resin. The resin was polymerized at 37 °C overnight followed by 60 °C for 48 h. Ultrathin sections were then prepared, stained, and examined under a Hitachi HT7800 microscope (Hitachi High-Tech Corporation, Tokyo, Japan) [11].

Formaldehyde-fixed samples were embedded in paraffin, sectioned by using a Leica RM2255 microtome (Leica Microsystems, Wetzlar, Germany), and stained with hematoxylin and eosin (H&E) for light microscopy (BX5; Olympus Corporation; Tokyo, Japan) coupled with a camera (H5500 L; Nikon Corporation; Tokyo, Japan) analysis according to conventional methods (Bancroft & Gamble, 2008 [12]). Villus height (V), crypt depth (C), and the villus height-to-crypt depth ratio (V/C) were measured.

### 2.4. Goblet Cell Counting

Goblet cells (GCs) were stained with Alcian Blue/Periodic Acid–Schiff (AB-PAS) and quantified according to established methods [12].

### 2.5. Activities of the Anti-Oxidant Enzymes

The activities of superoxide dismutase (SOD), catalase (CAT), total antioxidant capacity (T-AOC), glutathione peroxidase (GSH-Px), and malondialdehyde (MDA) content in the intestinal mucosa were measured using commercial kits (Nanjing Jiancheng Bioengineering Institute, Nanjing, China), following the manufacturer’s instructions. The assays were performed based on established spectrophotometric principles [13,14].

### 2.6. mRNA Expression Analysis

Gene expression levels of IL2, IL4, IFN-γ, and CD8 in the mucosal tissues of the duodenum, jejunum, and ileum were quantified using real-time PCR. β-Actin was used as the reference gene. RNA extraction, cDNA synthesis, and qPCR were performed following the Minimum Information for Publication of Quantitative Real-Time PCR Experiments (MIQE) guidelines [15] to ensure reproducibility. The 2^−ΔΔCT^ method was used for data analysis [16]. Primer sequences are listed in Table 2.

### 2.7. Immunohistochemical Staining

Protein expression of zonula occludens-1 (ZO-1), mucin 2 (Muc2), and occludin in the SI was detected via immunohistochemistry using standardized protocols [17]. Fixed samples were embedded in paraffin and sectioned by using a Leica RM2255 microtome (Leica Microsystems, Wetzlar, Germany) at 5 μm thickness. Following antigen retrieval, sections were incubated with primary antibodies: rabbit anti-Muc2 (1:100; abcam, ab76774), mouse anti-ZO-1 (1:100; Cell Signaling Technology, Danvers, MA, USA), and mouse anti-occludin (1:150; Cell Signaling Technology, USA). The optical density (OD) of the stained areas was quantified using Image J software (version 1.53k, National Institutes of Health, Bethesda, MD, USA).

### 2.8. Statistical Analysis

All data were analyzed using SPSS 18.0 software (SPSS Inc., Chicago, IL, USA) by an independent *t* test. The data are presented as Mean ± SE (standard error). Statistical difference was set at *p* < 0.05.

## 3. Results

### 3.1. Development of Small Intestine

No significant differences were observed in absolute SI length or weight between groups (*p* > 0.05). However, the length-to-body weight ratio of the duodenum and jejunum was higher in LBW piglets (*p* < 0.05) (Table 3).

### 3.2. Intestinal Morphology

**Figure 1:**
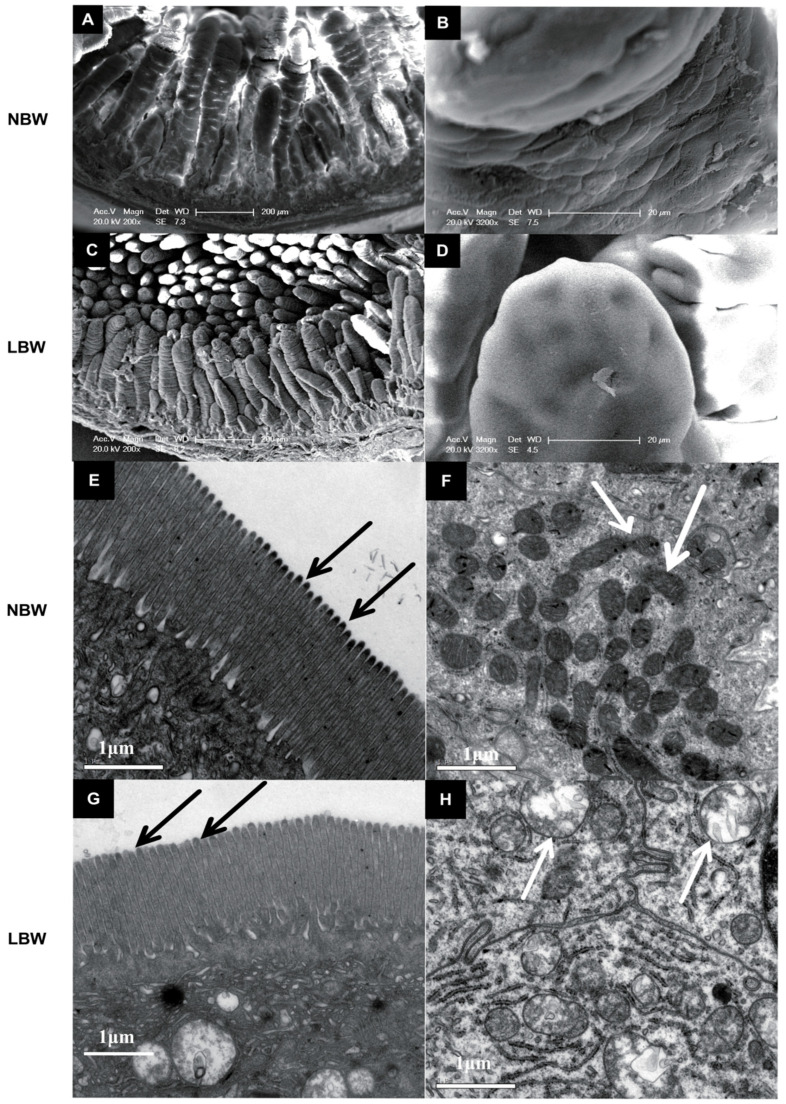
Effects of birth weight on the intestinal morphology and the interior structure of the intestine in Meishan pigs. (**A**,**B**,**E**,**F**) are pictures of the intestinal villi of normal-birth-weight (NBW) Meishan piglets, while the intestinal villus of low-birth-weight (LBW) piglets are shown in (**C**,**D**,**G**,**H**). The scale bar is 200 μm in (**A**) and (**C**), is 20 μm in (**B**) and (**D**) and is 1 μm in (**E**–**H**). Arrows indicate the mitochondria in panels (**F**) and (**H**).

LBW piglets exhibited decreased villus height (*p* < 0.01), villus height-to-crypt depth ratio (*p* < 0.001), and villus surface area (*p* < 0.05) in the jejunum, along with reduced villus height and V/C ratio in the ileum (*p* < 0.001) (Table 4). Villi appeared irregular and shortened with reduced surface area in LBW individuals. Microvilli were similar between groups, but mitochondrial swelling was observed in LBW piglets (Figure 1).

### 3.3. Goblet Cell Numbers and Activity of the Anti-Oxidant Enzymes

Goblet cell numbers were reduced in the jejunum (*p* < 0.05) and duodenum (*p* < 0.01) of LBW piglets (Table 4). Catalase activity was lower in the jejunum (*p* < 0.01), while glutathione peroxidase, catalase, and total antioxidant capacity were reduced in the ileum (*p* < 0.05) (Table 5).

### 3.4. Gene Expression of CD8 and Cytokines

Gene expression of CD8, IFNγ, and IL4 was downregulated in the jejunum and ileum of LBW piglets (*p* < 0.01), whereas gene expression of IL2 in the jejunum and IL4 in the ileum were upregulated (*p* < 0.01) (Table 6).

### 3.5. Expression of Mucin2 and Tight Junction Proteins

Protein expression of MUC2 was downregulated in the duodenum (*p* < 0.01) and ileum (*p* < 0.05) of LBW piglets (Table 7). Protein expression of ZO-1 was reduced across all SI segments (*p* < 0.05) (Table 7), and protein expression of occludin was also decreased (*p* < 0.05) in LBW piglets (Table 7). Expression of MUC2 and tight junction proteins is shown in the Supplementary Material.

## 4. Discussion

This study provides a comprehensive analysis of the impact of low birth weight (LBW) on intestinal barrier function in Meishan neonatal piglets, a breed known for its lower pre-weaning mortality despite reduced birth weight. To our knowledge, this is the first investigation to systematically compare intestinal mucosal immunity between LBW and normal-birth-weight (NBW) Meishan neonates, evaluating multiple aspects including morphology, antioxidant capacity, immune cell distribution, cytokine expression, and junctional protein integrity. A particularly noteworthy finding is the preservation of microvillus structure in LBW piglets, which stands in contrast to previous reports in LBW commercial breeds such as Duroc × (Landrace × Yorkshire) (Duroc × DLY) piglets, and may represent a key factor explaining the high survival rate of Meishan piglets despite their lower birth weight.

The increased small intestinal length-to-body weight ratio observed in LBW piglets may reflect a compensatory mechanism to enhance nutrient absorptive surface area, consistent with findings in intrauterine growth restriction (IUGR) models in commercial breeds [18]. However, the morphological alterations-including shortened and irregular villi, reduced villus surface area, and mitochondrial swelling-suggest structural and metabolic stress in the intestinal epithelium of LBW individuals. These structural deficits are likely to impair nutrient absorption and barrier function, as villus height and architecture are critical determinants of digestive efficiency [19]. Notably, and unlike what has been reported in LBW Duroc × DLY piglets, the microvillus structure remained completely intact in LBW Meishan neonates. This preservation of microvillus integrity may be of paramount importance, as microvilli dramatically increase the apical surface area of enterocytes and play crucial roles in nutrient digestion, absorption, and host–microbe interactions [8]. The maintained microvillus structure could therefore serve as a critical adaptive feature that supports nutrient assimilation and overall intestinal health in LBW Meishan piglets, potentially contributing to their remarkable survival rates.

The fundamental difference in microvillus resilience between Meishan and commercial breeds can be primarily attributed to inherent genetic factors. This premise is strongly supported by a recent authoritative review which synthesizes evidence that Chinese indigenous pig breeds, such as Meishan, possess distinct genetic advantages in intestinal development, including superior villus architecture, enhanced barrier function, and more robust responses to dietary and environmental challenges, compared to Western commercial breeds [20]. These breed-specific adaptations, potentially involving enhanced expression of cytoskeletal regulators, provide a compelling explanation for the preserved microvillus structure observed in LBW Meishan piglets in our study. Although the observed mitochondrial swelling indicates cellular stress, it did not appear to compromise microvillus integrity. These results suggested distinct mechanisms of cellular protection exist in Meishan piglets. The structural integrity of microvilli is critically dependent on actin-binding proteins such as villin and ezrin, which regulate cytoskeletal assembly and stability [21]. We speculate that a breed-specific expression or function of these and other cytoskeletal regulators could underlie the observed preservation of microvillus structure in LBW Meishan piglets. Future studies specifically measuring the expression and phosphorylation status of villin, ezrin, and other key structural proteins are needed to validate this hypothesis and define the precise molecular mechanisms. Further investigation into these protective mechanisms in Meishan pigs could provide valuable insights for improving survival in other swine breeds and possibly even in human neonates.

A key finding of this study is the reduction in goblet cell numbers and concomitant decrease in MUC2 expression in LBW piglets. Goblet cells and mucins constitute the first line of defense against luminal pathogens and toxins [22]. The decreased mucin production may predispose LBW piglets to increased intestinal permeability and inflammation, as reported in other IUGR models [23]. Interestingly, recent evidence suggests that goblet cell maturation and mucin secretion can be modulated by postnatal nutrition and gut microbiota [24]. Therefore, nutritional interventions aimed at stimulating mucin production—such as supplementation with threonine, probiotics, or prebiotics—may help restore mucosal integrity in LBW neonates [25].

The observed downregulation of tight junction proteins (ZO-1 and occludin) in LBW piglets further underscores the vulnerability of the intestinal barrier. Tight junctions play a crucial role in maintaining epithelial permeability and preventing antigen translocation [26]. Our results are consistent with studies in other porcine models showing that LBW or IUGR is associated with reduced expression of junctional proteins [27]. Furthermore, our results showed that LBW Meishan piglets exhibited impaired barrier function, as evidenced by downregulated tight junction proteins. It is important to note that the expression of these proteins and innate immune factors undergoes significant maturation during the first days of life in normal piglets [28]. The deficits we observed at birth may therefore indicate a delayed or compromised initiation of this crucial developmental program due to low birth weight. Recent research has highlighted the role of certain bioactive compounds, such as curcumin, berberine, and short-chain fatty acids, in enhancing tight junction assembly via modulation of signaling pathways like adenosine monophosphate-activated protein kinase (AMPK) and nuclear factor kappa-light-chain enhancer of activated B cells (NF-κB) [29,30]. These findings suggest that early nutritional or pharmacological intervention could mitigate barrier defects in LBW neonates.

Antioxidant capacity was also compromised in LBW piglets, as evidenced by reduced activities of CAT, GSH-Px, and T-AOC in the jejunum and ileum. Oxidative stress is increasingly recognized as a key contributor to intestinal injury in neonates, particularly under conditions of intrauterine growth restriction [7,31]. The mitochondrial swelling observed in our study may further exacerbate reactive oxygen species (ROS) production and impair cellular energy metabolism, creating a vicious cycle of oxidative damage and barrier dysfunction [32]. Recent studies suggest that antioxidant supplementation—for example, with vitamin E, selenium, or N-acetylcysteine—can alleviate intestinal oxidative stress and improve barrier function in piglets [33].

The dysregulated expression of cytokines and immune markers (CD8, IL2, IL4, IFNγ) indicates an imbalanced mucosal immune response in LBW piglets. The shift in T helper 1/T helper 2 (Th1/Th2) cytokine expression suggests a perturbed immune environment, which could influence both innate and adaptive intestinal immunity [34]. Similar immune dysregulation has been reported in human and animal models of LBW, often associated with an increased risk of inflammatory bowel disease or infection [35]. Interestingly, emerging research emphasizes the role of early-life microbial colonization in shaping immune programming, indicating that probiotic or synbiotic supplementation may help correct immune imbalances in LBW neonates [36].

Despite these deficits, Meishan piglets may possess certain advantages that mitigate the long-term impact of LBW. The preserved microvillus integrity, in particular, may serve as a fundamental protective factor that maintains absorptive function even in the face of other intestinal challenges. This unique characteristic, combined with the richer nutrient profile of Meishan sow milk (higher fat and calcium content), may support intestinal maturation and repair [37]. Moreover, the relatively small weight difference (−200 g) between LBW and NBW groups in this study may have limited the extent of intestinal compromise. It should be noted that the sample size in this study, while consistent with similar physiological investigations in large animal models, may limit the broad generalizability of the conclusions. Future studies should consider larger sample sizes and broader weight disparities to better capture the full spectrum of LBW-related intestinal dysfunction.

A critical point of consideration is the potential role of the maternal intrauterine environment in shaping the intestinal phenotype of the neonates. Although the piglets in this study were euthanized prior to suckling, thereby eliminating the influence of sow’s milk, they were nonetheless exposed to the in utero conditions provided by the sow throughout gestation. It is well-established that maternal factors such as systemic inflammation, exposure to endotoxins or mycotoxins (e.g., deoxynivalenol, DON), and oxidative stress can adversely affect fetal development, a concept often referred to as ‘fetal programming’ [38,39]. Notably, DON has been specifically shown to compromise intestinal integrity and trigger inflammation in sows, which could potentially be transmitted to the fetus, affecting its intestinal development [40]. The observed mitochondrial swelling and reduced antioxidant capacity in the LBW piglets could, for instance, be a reflection of a pro-oxidant intrauterine environment. Furthermore, the dysregulated cytokine profile and impaired barrier function might stem not only from the piglets’ low birth weight itself, but also from programming by maternal immune signals [41]. It is worth noting that the dietary tryptophan level (0.14%) used in this study was lower than that typically used in Western commercial sow diets (e.g., 0.18%). This formulation was based on the established nutritional standards for Meishan pigs, which may reflect breed-specific differences in nutrient metabolism and requirements. Further research is needed to clarify whether Meishan pigs have a lower tryptophan requirement and how this might influence sow performance and offspring development. While all sows in this study were clinically healthy and managed under the same conditions, we cannot rule out undetected subclinical variations in their health status that might have contributed to the differential development of their offspring. Future studies that directly correlate maternal blood biomarkers (e.g., inflammatory cytokines, oxidative stress markers) with neonatal intestinal outcomes would be invaluable in elucidating these transgenerational effects.

In conclusion, while LBW Meishan piglets exhibit clear impairments in several aspects of intestinal barrier function, the preservation of microvillus structure represents a remarkable adaptive feature that may significantly contribute to their enhanced survival rate compared to commercial breeds. This structural resilience, possibly resulting from genetic selection or breed-specific adaptations, is strongly associated with improved neonatal intestinal health. Understanding this trait suggests new avenues for improving survival in low-birth-weight neonates across species. These findings not only enhance our understanding of intestinal adaptation in LBW neonates but also underscore the importance of breed-specific management strategies to improve survival and health outcomes.

## 5. Conclusions

Low-birth-weight Meishan neonatal piglets exhibited alterations in intestinal barrier function, including structural changes in villus morphology, reduced antioxidant capacity, decreased goblet cell numbers, dysregulated cytokine expression, and downregulation of tight junction proteins. In striking contrast to these impairments, and in clear distinction to what is typically observed in commercial breeds, the microvillus structure remained completely intact in the small intestine of LBW Meishan neonates. This preservation of microvillus integrity represents a fundamental characteristic differentiating Meishan piglets and is identified as a critical adaptive mechanism that likely underpins their enhanced neonatal survival. These findings provide valuable insights into breed-specific intestinal adaptations and highlight the microvillus as a key structural determinant of neonatal resilience, suggesting new avenues for improving survival outcomes in low-birth-weight neonates across species.

## Figures and Tables

**Table 1:** Composition and nutrient levels of the diets of the sows of Meishan pigs (Dry matter basis; %).

Ingredients	Content (%)	Nutrient Levels ^§^	Content (%)
Corn	44.75	DE (kcal/kg) CP Lysine Threonine Tryptophan Crude Fiber Starch Ca Ap	2987.58 13.95 0.69 0.46 0.14 5.5 30 0.96 0.48
Soybean meal	13.60		
Wheat bran	27.8		
Soybean oil	4.50		
Wheat fiber	2.554		
Soybean fiber	1.10		
Corn fiber	0.96		
NaCl	0.40		
Choline	0.14		
CaCO_3_	1.24		
CaHpO_3_	1.99		
Vitamin premix ^†^	0.25		
Mineral premix ^‡^	0.50		
Lysine	0.10		
Threonine	0.10		

^†^ One kg of Vitamin premix contained the nutrients as following: Vitamin A 2,000,000 IU, Vitamin D3 400,000 IU, Vitamin E 20,000 IU, Vitamin K_3_, 600 mg; Vitamin B_12_, 8 mg; Riboflavin, 2000 mg; Niacin, 8000 mg; Pantothenic acid, 6000 mg. ^‡^ The mineral premix provided the following per kg of complete diet: Zn, 100 mg (as ZnSO_4_); Fe, 100 mg (as FeSO_4_); Mn, 40 mg (as MnSO_4_); Cu, 10 mg (as CuSO_4_); I, 0.3 mg (as KI); Se, 0.3 mg (as Na_2_SeO_3_).^§^ DE, digestible energy, Cp, crude protein, Ap, available phosphorus. DE was calculated in reference to Nutrition parameters and Feeding Standard for Animals, crude fiber, starch, and tryptophan were calculated based on typical nutrient composition of the ingredients, while the other nutrient levels were measured values. Amino acid values (Lysine, Threonine) represent total content.

**Table 2:** Primer sequences of genes for Real-time PCR.

Gene	Accession No.	Sequence (5′to 3′)	Length, bp
*β-actin*	XM 003357928	F TGCGGGACATCAAGGAGAAG R AGTTGAAGGTGGTCTCGTGG	216
*CD8*	NM_001001907.1	F AGCATTTGGGCCTCTCTTCC R ACTTACTGCATTGCCTCCCC	140
*IL2*	NM 213861.1	F TGCACTAACCCTTGCACTCA R GCAATGGCTCCAGTTGTTTCT	83
*IL4*	NM_214123	F CTCCCAACTGATCCCAACCC R TGCACGAGTTCTTTCTCGCT	134
*IFN-γ*	NM 213948.1	F ACCAGGCCATTCAAAGGAGC R CGAAGTCATTCAGTTTCCCAGAG	90

**Table 3:** Effects of birth weight on the development of the SI in Meishan piglets †.

Item	Groups		*p*-Value
	NBW	LBW	
body weight (kg)	0.85 ± 0.06	0.65 ± 0.02	0.028
length of duodenum (cm)	11.58 ± 1.22	11.70 ± 0.89	0.938
weight of duodenum (g)	0.89 ± 0.11	0.80 ± 0.03	0.460
length of jejunum (cm)	194.40 ± 9.43	176.64 ± 6.90	0.167
weight of jejunum (g)	11.58 ± 1.26	8.99 ± 0.72	0.113
length of ileum (cm)	135.00 ± 10.78	117.76 ± 4.60	0.179
weight of ileum (g)	11.26 ± 1.40	7.96 ± 0.37	0.076
length of duodenum: BW (cm/kg)	13.56 ± 1.07	18.19 ± 1.60	0.043
weight of duodenum: BW (g/kg)	1.03 ± 0.12	1.24 ± 0.07	0.187
jejunum length: BW (cm/kg)	230.48 ± 8.44	273.88 ± 13.40	0.025
jejunum weight: BW (g/kg)	13.48 ± 0.67	13.83 ± 0.90	0.764
ileum length: BW (cm/kg)	159.13 ± 7.05	182.59 ± 8.94	0.073
ileum weight: BW (g/kg)	13.07 ± 0.85	12.38 ± 0.86	0.587

† Values are means ± SE, *n* = 6. BW, body weight; NBW, normal birth weight; LBW, low birth weight. *p* < 0.05 mean values within the same line are significantly different.

**Table 4:** Effects of birth weight on the villus morphology and the goblet cell number in the SI of Meishan piglets †.

Sites	Items	Groups		*p*-Value
		NBW	LBW	
duodenum	Villus length (μm)	352.34 ± 3.71	346.85 ± 12.68	0.696
	Crypt depth (μm)	37.56 ± 1.31	39.24 ± 0.85	0.315
	Villus width (μm)	53.23 ± 1.27	51.83 ± 2.86	0.665
	V/C	9.41 ± 0.24	8.83 ± 0.17	0.082
	Villus surface area (mm^2^)	0.030 ± 0.001	0.028 ± 0.002	0.521
	number of goblet cells	28.37 ± 2.46	20.41 ± 1.00	0.028
jejunum	Villus length (μm)	496.68 ± 4.78	373.25 ± 16.92	0.001
	Crypt depth (μm)	49.50 ± 0.24	41.12 ± 1.40	0.003
	Villus width (μm)	52.07 ± 1.37	47.91 ± 3.65	0.334
	V/C	10.08 ± 0.10	9.08 ± 0.15	<0.001
	Villus surface area (mm^2^)	0.041 ± 0.001	0.029 ± 0.003	0.018
	numbers of goblet cells	26.28 ± 0.94	19.98 ± 1.43	0.006
ileum	Villus length (μm)	475.01 ± 7.38	394.35 ± 5.52	<0.001
	Crypt depth (μm)	50.13 ± 0.46	55.58 ± 1.83	0.039
	Villus width (μm)	61.50 ± 2.15	65.53 ± 2.27	0.233
	V/C	9.49 ± 0.10	7.17 ± 0.18	<0.001
	Villus surface area (mm^2^)	0.046 ± 0.002	0.041 ± 0.002	0.121
	numbers of goblet cells	19.81 ± 0.79	17.89 ± 0.89	0.148

† Values are means ± SE, *n* = 6. V/C, the ration of villus length versus crypt depth; NBW, normal birth weight; LBW, low birth weight. *p* < 0.05, *p* < 0.01, *p* < 0.001 mean values within the same line are significantly differ.

**Table 5:** Effects of birth weight on the activity of the anti-oxidant enzymes in the mucosa of SI in Meishan piglets †.

Sites	Items	Groups		*p*-Value
		NBW	LBW	
jejunum	CAT(U/mg prot)	4.48 ± 0.55	2.04 ± 0.30	0.005
	GSH-px (U)	26.77 ± 2.98	16.42 ± 4.89	0.109
	MDA (nmol/mg prot)	20.56 ± 1.22	21.87 ± 0.93	0.417
	SOD (U/mg prot)	5.59 ± 0.11	5.55 ± 0.03	0.742
	T-AOC (mmol/g)	1.23 ± 0.02	1.24 ± 0.01	0.891
ileum	CAT(U/mg prot)	5.54 ± 0.62	3.91 ± 0.31	0.047
	GSH-px (U)	26.96 ± 4.67	11.18 ± 1.25	0.025
	MDA (nmol/mg prot)	16.77 ± 0.70	14.56 ± 0.88	0.086
	SOD (U/mg prot)	5.49 ± 0.01	5.50 ± 0.01	0.085
	T-AOC (mmol/g)	1.29 ± 0.01	1.19 ± 0.03	0.025

† Values are means ± SE, *n* = 6. CAT, catalase; GSH-px, glutathione peroxidase; MDA, malondialdehyde; SOD, superoxide dismutase; T-AOC, total antioxidant capability; NBW, normal birth weight; LBW, low birth weight. *p* < 0.05, *p* < 0.01 mean values within the same line are significantly different.

**Table 6:** Effects of birth weight on the relative gene expression of CD8 and cytokines in SI of Meishan piglets †.

Sites	Items	Groups		*p*-Value
		NBW	LBW	
jejunum	CD8	1.00 ± 0.01	0.67 ± 0.07	0.002
	IL2	1.00 ± 0.04	2.19 ± 0.08	<0.001
	IL4	1.00 ± 0.02	0.37 ± 0.01	<0.001
	IFNγ	1.00 ± 0.02	0.13 ± 0.01	<0.001
ileum	CD8	1.00 ± 0.04	0.24 ± 0.01	0.002
	IL2	1.00 ± 0.03	0.22 ± 0.003	<0.001
	IL4	1.00 ± 0.04	1.30 ± 0.06	0.004
	IFNγ	1.00 ± 0.02	0.11 ± 0.004	<0.001

† Values are means ± SE, *n* = 6. IL2, interleukin 2; IL4, interleukin 4; IFNγ, interferon γ; NBW, normal birth weight; LBW, low birth weight. *p* < 0.05, *p* < 0.01, *p* < 0.001 mean values within the same line are significantly different.

**Table 7:** Effects of birth weight on the protein expression (optical density of the stained areas) of tight junctions in SI of Meishan piglets †.

Sites	Items	Groups		*p*-Value
		NBW	LBW	
duodenum	MUC2	0.26 ± 0.006	0.16 ± 0.009	0.001
	occludin	0.25 ± 0.010	0.200 ± 0.026	0.038
	ZO-1	0.25 ± 0.010	0.22 ± 0.015	0.034
jejunum	MUC2	0.23 ± 0.015	0.20 ± 0.023	0.336
	occludin	0.20 ± 0.010	0.17 ± 0.015	0.034
	ZO-1	0.24 ± 0.015	0.18 ± 0.010	0.006
ileum	MUC2	0.27 ± 0.015	0.20 ± 0.030	0.027
	occludin	0.23 ± 0.025	0.17 ± 0.015	0.035
	ZO-1	0.26 ± 0.025	0.18 ± 0.015	0.009

† Values are means ± SE, *n* = 6. MUC2, mucin2; ZO-1, Zonula occludens-1; NBW, normal birth weight; LBW, low birth weight. *p* < 0.05, *p* < 0.01 mean values within the same line are significantly different.

## Data Availability

The data from the study are available from the corresponding authors upon reasonable request.

## Supplementary Materials

The following supporting information can be downloaded at: https://www.mdpi.com/article/10.3390/ani15213085/s1.
