# Supplementary figures and images for: Microvillus in LBW Meishan Piglets Preserved Microvillus Integrity Alongside Impaired Intestinal Barrier Function in Low-Birth-Weight Meishan Neonatal Piglets
Source: Animals (Basel). 2025 Oct 24;15(21):3085. doi: 10.3390/ani15213085 (PMC12610649; doi:10.3390/ani15213085)

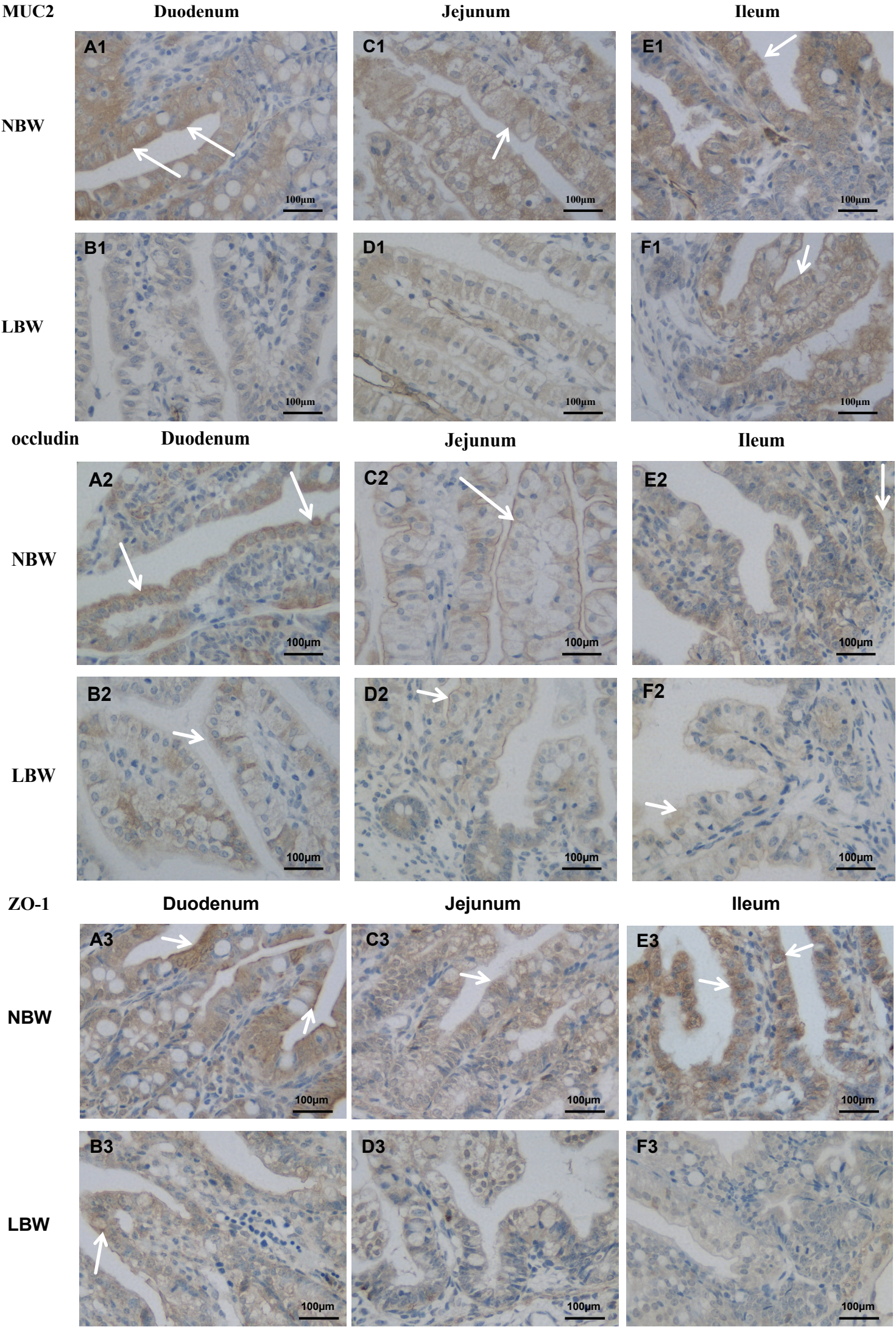

Supplement: Supplementary file 1 [file animals-15-03085-s001.zip › animals-3880305-supplementary.pdf]
